# The multifunctional roles of autophagy in the innate immune response: Implications for regulation of transplantation rejection

**DOI:** 10.3389/fcell.2022.1007559

**Published:** 2022-12-21

**Authors:** Kunli Zhang, Qiuyan Huang, Laru Peng, Sen Lin, Jie Liu, Jianfeng Zhang, Chunling Li, Shaolun Zhai, Zhihong Xu, Sutian Wang

**Affiliations:** ^1^ Institute of Animal Health, Guangdong Academy of Agricultural Sciences, Guangdong Provincial Key Laboratory of Livestock Disease Prevention Guangdong Province, Scientific Observation and Experiment Station of Veterinary Drugs and Diagnostic Techniques of Guangdong Province, Ministry of Agriculture and Rural Affairs, Guangzhou, China; ^2^ State Key Laboratory of Livestock and Poultry Breeding, Guangdong Key Laboratory of Animal Breeding and Nutrition, Institute of Animal Science, Guangdong Academy of Agricultural Sciences, Guangzhou, China; ^3^ Guangzhou Laboratory, Guangzhou International BioIsland, Guangzhou, China; ^4^ Sericultural & Agri-Food Research Institute, Guangdong Academy of Agricultural Sciences, Guangzhou, China; ^5^ Guangdong Yantang Dairy Co, Ltd, Guangzhou, China; ^6^ Maoming Branch, Guangdong Laboratory for Lingnan Modern Agriculture, Maoming, China

**Keywords:** organ transplantation, graft rejection, autophagy, innate immune response, oxidative stress, cell death

## Abstract

Organ transplantation is the main treatment for end-stage organ failure, which has rescued tens of thousands of lives. Immune rejection is the main factor affecting the survival of transplanted organs. How to suppress immune rejection is an important goal of transplantation research. A graft first triggers innate immune responses, leading to graft inflammation, tissue injury and cell death, followed by adaptive immune activation. At present, the importance of innate immunity in graft rejection is poorly understood. Autophagy, an evolutionarily conserved intracellular degradation system, is proven to be involved in regulating innate immune response following graft transplants. Moreover, there is evidence indicating that autophagy can regulate graft dysfunction. Although the specific mechanism by which autophagy affects graft rejection remains unclear, autophagy is involved in innate immune signal transduction, inflammatory response, and various forms of cell death after organ transplantation. This review summarizes how autophagy regulates these processes and proposes potential targets for alleviating immune rejection.

## 1 Introduction

As the main treatment for end-stage organ failure, organ transplantation is an effective way to rescue patients in a clinical emergency. Besides organ shortages, immune rejection, immune tolerance, and early graft failure are the most concerning problems (Stolp et al., 2019). The immune system’s discrimination between self and non-self is a necessary protection mechanism (Abou-Daya and Oberbarnscheidt, 2021). It is widely acknowledged that the essence of transplantation rejection is the immune response of the recipient’s immune system to a donor graft. Since T cells and B cells are considered the main effector cells of transplantation, the role of adaptive immunity in transplantation rejection has always been a hotspot (Wood et al., 2012; Romano et al., 2017; Schmitz et al., 2020; Asano et al., 2021; Otsuka et al., 2021). However, a donor graft first causes inflammation and tissue damage through activation of innate immunity, which further trigger adaptive immune responses. It is innate immune receptor, which first recognizes allogeneic non-self and trigger innate immune responses, that is responsible for the initiation of adaptive immunity (Abou-Daya and Oberbarnscheidt, 2021; Wang B. et al., 2021). After the organ transplantation, a series of factors that target transplanted organs can result in innate immune responses, such as surgical mechanical injury, ischemia reperfusion injury (IRI), inflammation, oxidative stress and death of donor cells in transplanted organs. The role of innate immune in transplantation rejection and survival of graft is receiving more attention in recent years (Ochando et al., 2019; Shepherd et al., 2021).

The immune functions of an organism have been strictly regulated to maintain homeostasis. When a body receives danger signals, it will timely initiate immune responses to remove the threat. Meanwhile, the body also needs to turn off the immune response in time to avoid damage. Autophagy is an evolutionarily conserved degradation system that degrades intracellular toxic substances or organelles to provide materials for physiological metabolism and maintain homeostasis. As research continues, a number of studies suggest that various physiological signals are closely linked to autophagy, such as inflammation, oxidative stress, organelle damage, cell death and immune signal transduction (Filomeni et al., 2015; Deretic, 2021). Moreover, autophagy also takes part in the immune response when a body is subjected to adverse stimuli (Jiang et al., 2019). On the one hand, autophagy can affect adaptive immunity by participating in antigen presentation and regulating adaptive immune cells’ activation, proliferation, and differentiation of adaptive immune cells (Puleston and Simon, 2014). On the other hand, autophagy is also involved in regulating innate immune signalling pathways through the degradation of inflammatory protein, oxidative stress intermediate molecules and the interaction with pattern recognition receptors (PRRs) (Germic et al., 2019). Since graft rejection is obviously influenced by innate immunity, which is partly regulated by autophagy, it is necessary to explore the role of autophagy in organ transplantation. Several studies have suggested that autophagy plays a decisive role in graft survival. The enhanced autophagy prolongs skin allograft survival in human myeloid dendritic cells (Lin et al., 2013). mTOR-mediated autophagy facilitates mouse cardiac allograft survival by enhancing the immunosuppressive function of myeloid-derived suppressor cells (Li J. et al., 2021). Increased expression of autophagy-related proteins (Beclin-1 and LC3) is essential for reducing the incidence of necrosis and rejection after liver transplantation (Degli Esposti et al., 2011). Here, we discuss the multifunctional roles of autophagy in graft rejection from the perspective of innate immunity. And we also offer a strategy for inhibiting the immune rejection response following graft transplantation.

## 2 Crosstalk between autophagy and pattern recognition receptor in organ transplantation

### 2.1 TLRs link inflammation with autophagy during graft transplants

In the past decades, considerable evidence indicates that the activation of the innate immune depends on PRRs, which can recognize all kinds of endogenous and exogenous danger signals (mainly including pathogen-associated molecular patterns (PAMPs) and damage-associated molecular patterns (DAMPs)). Most PRRs of vertebrates are classified into five types which are Toll-like receptors (TLRs), nucleotide oligomerization domain-like receptors (NLRs), C-type lectin receptors (CLRs), retinoic acid-inducible gene-I like receptors (RLRs), and absent in melanoma-2 like receptors (ALRs). These receptors recognize and bind their respective ligands and then initiate downstream signalling pathways. The transplant surgery inevitably leads to tissue damage, oxidative imbalance or bacterial translocation, which acts as PAMP and DAMP to trigger the recruitment and activation of inflammatory cells (Ochando et al., 2019). In this process, the PRRs activation is closely linked to autophagy, which always shares similar signalling pathways. Many experimental and clinical research findings revealed that TLRs are instrumental in the inflammatory response and immune rejection (Braza et al., 2016). Under the influence of surgical incisions and intestinal stress, clinical liver and intestinal transplantation surgery cannot avoid the translocation of commensal bacteria, which will cause an innate immune response by activating TLRs (Alegre et al., 2008). In addition, the transplantation-associated IRI can also induce upregulation of DAMPs, such as high-mobility group box chromosomal protein 1 (HMGB1) and heat shock protein (HSP) fibrinogen, hyaluronan and biglycan (Wu et al., 2010; Patel et al., 2012). These substances can be recognized and bound by TLRs, and then activate TLRs-MyD88/TRIF signalling pathways to cause pro-inflammatory responses, which contribute to chronic graft dysfunction (Wang et al., 2010). Activation of TLRs contributes to the development of graft rejection which is attributed to the production of various pro-inflammatory cytokines (including IL-1β, IL-6, and TNF-α) and nitroxidation intermediates (including iNOS, NO, O_2_) (Kaczorowski et al., 2007). Meanwhile, autophagy is also considered an immune effector that can be activated by TLRs signalling (Zhang K. et al., 2021). Both TLRs-MyD88 and TLRs-TRIF signalling pathways are involved in regulating autophagy activity. The activation of TLRs signalling can enhance the interaction between Beclin-1 and MyD88/TRIF and then promotes the dissociation of coiled-coil moesin-like BCL2-Interacting Protein (Beclin-1) and B cell lymphoma 2 (Bcl-2), leading to autophagy activation (Shi and Kehrl, 2008). Moreover, the activation of TNF receptor associated factor 6 (TRAF6) induces the K63-linked polyubiquitination of Beclin-1, which enhances phosphatidylinositol 3 kinase catalytic domain (PI3KC) activity and promotes autophagy initiation (Lee et al., 2018). Yang et al. suggested that enhancing autophagy could alleviate renal IRI *via* TLR4/MyD88/ERK/mTOR signalling after renal transplantation (Yang et al., 2020). A similar result also showed that the inhibited autophagy contributed to aggravating hepatic IRI *via* Mitogen-Activated Protein Kinase/mammalian target of rapamycin (MAPK/mTOR) signalling (Xu et al., 2018). However, Xu et al. reported an opposite result that overactivated autophagy aggravates the cerebral IRI *via* MAPK/ERK/mTOR (Xu D. et al., 2021). It should be noted that the results on the effects of different autophagy stages activities on cerebral IRI were lacking in this study. But we cannot deny that autophagy is a double-edged sword in maintaining homeostasis, and the specific mechanism of these differences needs to be further studied.

### 2.2 Other PRRs mediated autophagy in graft IRI

**FIGURE 1:**
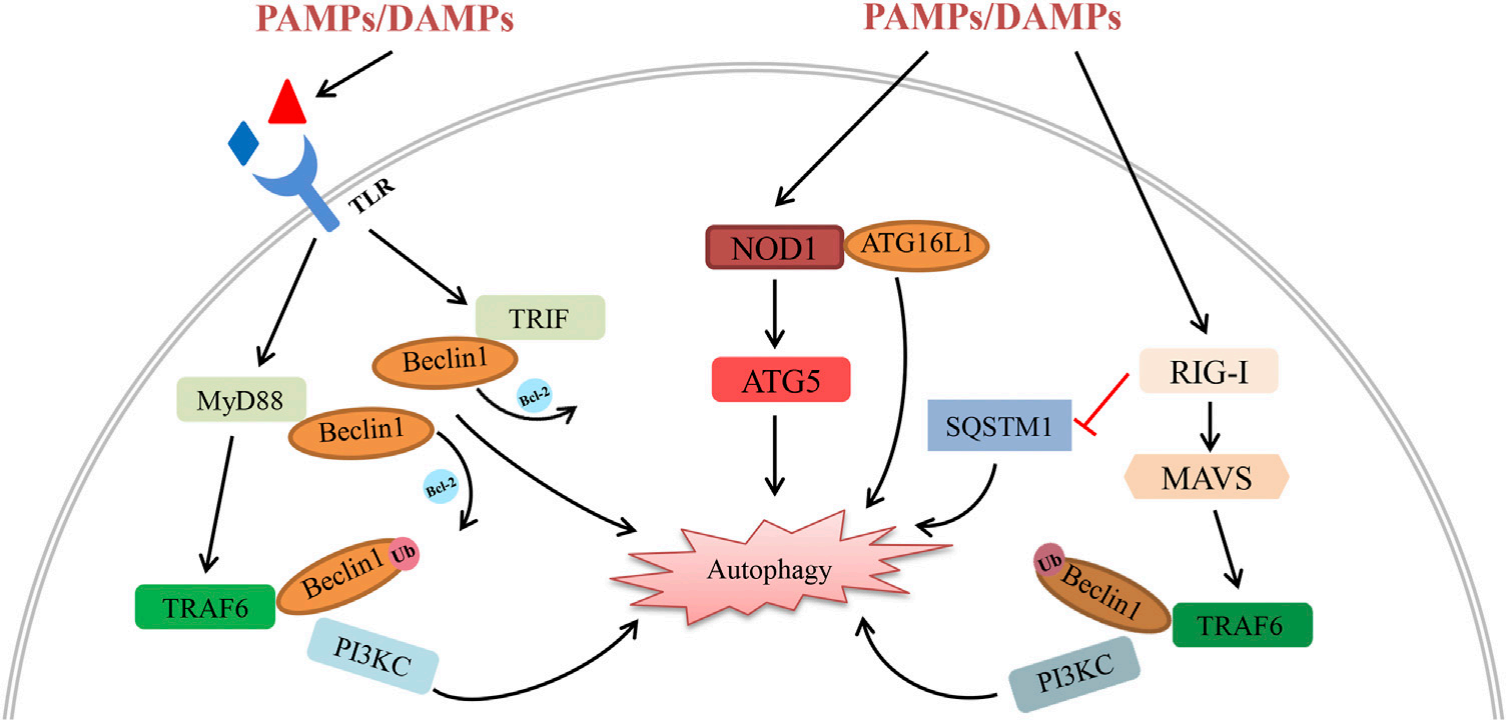
Schematic diagram of the interplay between autophagy and PRRs (including their downstream signalling molecules) in organ transplantation. All kinds of PAMPs/DAMPs can be recognized by PRRs, activating their downstream signalling pathway following organ transplantation. (1) PAMPs/DAMPs directly bind to TLRs and activate the MyD88-TRAF6 and TRIF pathways. Activation of TRAF6 induces the K63-linked polyubiquitination of Beclin-1, promoting autophagy degradation by enhancing PI3KC activity. In addition, the interaction between Beclin-1 and MyD88/TRIF induces the dissociation of Beclin-1 and Bcl-2, leading to autophagy initiation. (2) Activation of NOD1 by reperfusion contributes to autophagic degradation *via* the interaction with ATG16L1 and ATG5. (3) On one hand, the expression of RIG-I decreases SQSTM1 and boosts autophagic degradation. On the other hand, activation of RIG-I triggers autophagy *via* the MAVS-TRAF6-Beclin-1 signalling axis.

NLR is an important cytoplasmic pattern recognition receptor recognizing PAMPs and DAMPs similar to TLRs. As a member of the NLRC (NOD-like receptor containing a CARD domain) family, Nucleotide-binding oligomerization domain-containing protein 1 (NOD1) has been proved to directly interact with and recruit autophagy related protein 16 like protein 1 (ATG16L1) to the plasma membrane to activate autophagy (Travassos et al., 2010). Xi et al. observed the expression of NOD1 and autophagy levels increased after Hepatic IRI. Inhibition of NOD1 could down-regulate ATG5 and Beclin-1 levels (Xi et al., 2019). It seemed that NOD1 might regulate autophagy by PI3K/AKT/mTORC1 signalling, but there is still lacked a specific interaction mechanism between NLRs and the autophagy system. RLRs are a new class of PRRs which mainly recognize viral RNA. Ordinarily, the activation of RLR is important for establishing innate antiviral immunity *via* interferon and pro-inflammatory cytokines induced by its downstream signal transduction. Retinoic acid-inducible gene I (RIG-I) is one of the most important members of the RLR family and can recognize short triphosphorylated dsRNA. The heterodimer complex of ATG5 and ATG12 can directly interact with RIG-I, melanoma differentiation-associated gene 5 (MDA5) and IPS-1, inhibiting their dissociation and negatively controlling RLR signal transduction (Jounai et al., 2007). Several studies have indicated the direct interaction between autophagy receptors and RLR family members (Lee et al., 2018; Xian et al., 2020; Hou et al., 2021). Activation of RIG-I can induce K63-linked polyubiquitination of Beclin-1 and affect sequestosome 1 (SQSTM1) accumulation, leading to autophagic degradation. Despite the fact that very few studies show the links between organ transplantation, RLR and autophagy, we believe they have important roles in common viral infections in transplant recipients, which deserve more attention. Although some progress has been made to indicate a crucial role for these PRRs in affecting graft IRI, regulating immune response, and recognizing all kinds of danger signals in organ transplantation, further study is needed to explore their distinct roles and specific mechanisms that regulate graft dysfunction (Figure 1).

## 3 Autophagy regulates inflammation response and oxidative stress in organ transplantation

In organ transplant surgery, mechanical injury, hypoxia, ischemia, and reperfusion injury have inevitably happened. The combined effects of these factors trigger the recipient’s innate immune recognition, then cause inflammation, injury and death in the cells of the graft tissue, which are involved in the innate immune effect mechanisms of transplantation rejection. Autophagy generally exerts a protective effect to regulate inflammatory response and stress injury to maintain cellular homeostasis when cells are under stress. Therefore, it is worth discussing the mechanisms by which autophagy regulates inflammation and oxidative stress after organ transplantation.

### 3.1 Graft inflammation and autophagy

The inflammasome is an important component of the innate immune system and is essential for inflammation initiation after recognizing various PAMPs or DAMPs. Saitoh et al. first discovered the loss of autophagy-related protein ATG16L1/ATG7 or inhibition of autophagy could activate inflammasome and increase IL-1β production (Saitoh et al., 2008). Over the past few years, many studies have proven autophagy can affect inflammation response by regulating inflammasome activity (Zhou et al., 2011; Liu, 2019; Mehto et al., 2019). The special mechanisms mainly relate to removing inflammasome activators (including damaged organelles, ROS) or degrading the inflammasome directly (Biasizzo and Kopitar-Jerala, 2020). Moreover, autophagy also has a direct impact on the transcription, processing and secretion of inflammatory factors. For example, autophagy can target and degrade pro-IL-1β, decreasing the secretion of mature IL-1β (Harris et al., 2011). By using autophagy activator rapamycin or autophagy inhibitor 3MA, Wang et al. indicated that autophagy could alleviate hepatic IRI injury *via* decreasing NOD-like receptor thermal protein domain associated protein 3 (NLRP3), IL-1β and IL-18 production (Wang et al., 2021b). Another study showed that hepatic IRI could increase PTEN-induced kinase 1 (PINK1) and Parkin RBR E3 ubiquitin-protein ligase (PARKIN) levels, which are necessary for mitophagy initiation. Furthermore, overexpression of PINK1 inhibited NLRP3 inflammasome activation and decreased IL-1β production during hepatic IRI *in vivo* (Xu et al., 2020b). Moreover, activating LKB1-AMPK-ULK1 signalling promoted myocardial autophagy, inhibiting inflammatory cytokine release and prolonging cardiac allograft survival (Chen et al., 2020). Similarly, ATG5-deficient mice are subjected to kidney IRI, which displays more severe sterile inflammation (Liu et al., 2012). Interestingly, another research group showed that ATG5 also participated in antibody-mediated rejection during kidney grafts, which suggested that Atg5-mediated autophagy could affect graft survival through multiple pathways (Cheng et al., 2021). Recently, a study suggested rapamycin, an autophagy activator, enhanced autophagy and alleviated corneal allograft rejection (Wei et al., 2022). Mechanistically, the enhanced autophagic turnover by rapamycin inhibited NLRP3 inflammasome, cleaved Casp-1(p10), and IL-1β through NLRP3 degradation. Additionally, the high-level NLRP3 would up-regulate V-ATPase D2 subunit, which gradually promoted the formation of autophagolysosomes to increase autophagy flux to limit the IR-induced inflammation in a Notch1-Hes1-independent manner (Wang et al., 2021c). These studies suggested autophagy helped to reduce inflammation after organ transplantation. However, autophagy plays a dual role in the regulation of inflammation. Interstitial fibrosis is a leading cause of chronic graft dysfunction. After renal transplantation, the enhanced ATG16L-dependent autophagic flux leads to renal interstitial fibrosis and chronic renal graft dysfunction through triggering endothelial-mesenchymal transition (EndMT) by NF-κB, IL-1β, IL-6 and TNF-α (Gui et al., 2021a; Gui et al., 2021b). Moreover, ATG5 also promotes IL-6 secretion in dendritic cells, which drives chronic heart allograft rejection after IRI (Solhjou et al., 2017). The role of autophagy in regulating inflammation-related graft rejection varies widely among tissues and organs and immune stages. More work still needs to explore the potential mechanisms by which autophagy and inflammation regulate graft rejection. Also, it is unclear whether these different results are due to the effects on apoptosis, which also can be influenced by autophagy. IFN-γ is a key regulator of the homeostasis of kidney transplant rejection and is closely linked to autophagy and inflammatory factors production (Hidalgo and Halloran, 2002). IFN-γ has been shown to have contradictory effects on the survival of the graft (Brandacher et al., 2007). Most studies on the effect of IFN-γ on graft rejection have focused on its effect on T cell activity. A recent study showed the interaction between autophagy and IFN-γ affected the prognosis of renal transplantation. However, the specific mechanism of the effect of autophagy-IFN signalling on organ transplantation rejection remains to be studied. With the spread of COVID-19 in recent years, the importance of autophagy in influencing lung inflammation and injury is widely concerned. A team has discovered that enhancing autophagy could decrease IRI-induced lung injury *via* the PI3K/Akt signalling pathway (Li J. et al., 2015). The increasing autophagy contributes to decreasing concentrations of IL-1β and TNF-α and the ratio of dead cells. In addition, the mucosal injury and bacterial translocation triggered by intestinal transplantation could induce a life-threatening systemic inflammatory response (Wang et al., 2020). Intestinal IRI increased levels of NLRP3, TNF-α, IL-6 and p62, as well as a decreased ratio of LC3-II/I. Adding autophagy activator rapamycin alleviated intestine damage and accumulation of inflammasome (Wang et al., 2019).

### 3.2 The interaction between graft oxidative stress and autophagy

During transplantation, a specific organ is subjected to blood supply arrest followed by a sudden hyperoxygenation at the reperfusion time. This process induces severe oxidative stress, which is one of the great causes of graft damage and death (Carcy et al., 2021). Moreover, cell death and tissue injury also contribute to activating the innate immune and leading to the production of the pro-inflammatory cytokine, reactive oxygen species (ROS) and nitric oxides (NO), whose effects are to trigger severe cellular stress followed by inflammatory cascades, and ultimately lead to failed organ transplants. To avoid further oxidative stress, the body can activate a series of defence responses to maintain physiological homeostasis, including autophagy. The inter-relational mechanism between autophagy and oxidative stress has recently been a hotspot. ROS can directly induce oxidative stress by regulating the activity of multiple upstream autophagy pathways, including AMP-activated Kinase (AMPK), mTOR, MAPK, and PI3K (Liu et al., 2015; Portal-Nunez et al., 2016; Zhang K. et al., 2021). In addition, ROS also can modify autophagy-related proteins to regulate autophagy activity (Lv et al., 2018; Zhang K. et al., 2021). There is plenty of evidence that transplantation-induced ROS can activate autophagy (Van Erp et al., 2017). Autophagy, in turn, regulates oxidative stress by removing damaged organelles and excess oxidizing intermediates (Larabi et al., 2020). Losses of autophagy-related proteins contribute to the accumulation of cellular ROS (Asano et al., 2017; Saxena et al., 2018). During transplant surgery, both hypoxia and hyperoxia can induce autophagy. The interaction between hypoxia-inducible factor-1α (HIF-1α) signalling and autophagy-related genes such as ATG2A, ATG14, and Beclin-1 contributes to hypoxia-induced autophagy in renal IRI (Bizargity and Schroppel, 2014; Fu et al., 2020; Li Q. et al., 2021). More importantly, hyperoxia- or oxidizing intermediates-induced protective autophagy (which means the autophagy that contributes to removing stimulus and maintaining homeostasis) improves graft longevity. Regulation of oxidative stress by autophagy during graft IR is best studied. Ischemia leads to ATP generation dysfunction and intracellular acidosis. These disorders break the osmotic equilibrium, which causes leukocyte infiltration of the graft after reperfusion (Cucchiari et al., 2016). Moreover, the reperfusion creates a hyperoxygenation environment and produces large amounts of ROS in just a few moments. As the most important energy supply structure, mitochondria mainly contribute to ROS production. Many researchers believe that ROS promote autophagy activation, and the activated autophagy contributes to clearing ROS. Removal of ROS by autophagy helps to avoid mitochondrial damage risk. Forkhead box O3 (FOXO3) signalling, Nuclear factor erythroid 2-related factor 2 (Nrf2) signalling and HIF-1α signalling are involved in this process (Li L. et al., 2015). While along with the development of autophagy research, the dynamics and roles of autophagy in different graft IR remain elusive. Studies have shown that the autophagy level is up-regulated in cardiac and renal IR but decreased in hepatic IR (Van Erp et al., 2017). Further studies showed that autophagy could ameliorate hepatic IRI but aggravate cardiac (Cursio et al., 2015; Decuypere et al., 2015; Ma et al., 2015; Wang et al., 2018; Xu T. et al., 2021). Comparing these different studies, we speculate that the distinct results might be attributed to cell types, injury levels, stress intensity and duration (Liu et al., 2013; Guo et al., 2015). It seems that moderate graft IR stress will trigger protective autophagy, which contributes to clearing ROS for maintenance of homeostasis. Under excessive graft IR stress, in turn, autophagy not only fails to promote ROS degradation but also aggravates graft injury, which may be attributed to excessive autophagosome formation or dysfunction of the autophagy/lysosomal degradation.

### 3.3 Autophagy affects the signals shared by graft inflammation and oxidative stress

Actually, a body’s inflammation response and oxidative stress always happen simultaneously following organ transplantation. The activation of inflammation response and oxidative stress shares some signalling nods, such as nuclear factor kappa-B (NF-κB), MAPK, HIF-1α, and NLRP3 (McGarry et al., 2018). Excessive accumulation of ROS can lead to mitochondrial dysfunction. The release of mitochondrial damage-associated molecular patterns (mtDAMPs) can activate cell surface receptors or intracellular receptors to initiate innate immune responses and subsequently promotes inflammatory gene expression (McGarry et al., 2018). Moreover, mitochondrial ROS is involved in assembling the inflammasomes, which boosts the activation of NLRP3-dependent IL-1β and IL-18 maturation (Zhou et al., 2011). Hypoxia-induced overproduction of ROS can affect HIF-1α signalling activity, which is involved in the production of inflammatory cytokines (Patten et al., 2010; Muz et al., 2012). Similarly, several studies indicated ROS could activate NLRP3 *via* NF-κB and MAPK signalling (An et al., 2019; Papaconstantinou, 2019). Therefore, it is necessary to survey the combined effect of inflammation and oxidative stress, which are important immune responses in transplantation rejection (Table 1). More importantly, as a key regulator in this process, autophagy deserves to be further studied. Most of the recent research just showed the effect of autophagy on inflammation gene expressions and oxidative stress levels. The potentially specific mechanism needs to be further explored.

**TABLE 1:** Regulation of inflammation response and oxidative stress in organ transplantation by autophagy.

Autophagy-related gene/signalling	Target	Function	References
ATG16L1/ATG7	NLRP3, IL-1β	Suppresses inflammasome formation and IL-1β production	Saitoh et al. (2008)
PINK1, PARKIN	NLRP3, IL-1β	Suppresses NLRP3 activation and decreased IL-1β production during hepatic IRI	Xu et al. (2020b)
ATG5	IL-1β, TNF-α	during kidney IRI	Liu et al. (2012)
mTOR	NLRP3, TNF-α, IL-6	Suppresses NLRP3 activation and decreases TNF-α and IL-6 production during intestinal IRI	Wang et al. (2019)
ATG2A, ATG14, Beclin-1	HIF-1α-BNIP3	Promotes autophagy and decreases ROS, and ameliorates renal IRI	(Bizargity and Schroppel, (2014); Fu et al. (2020); Li et al. (2021b))
MEK/ERK/mTOR	ROS	Promotes autophagy, decreases ROS and ameliorates hepatic IRI	Wang et al. (2018)
Beclin-1	ROS	Promotes autophagy, decreases ROS and ameliorates renal IRI	Xu et al. (2021b)

## 4 Cell death and autophagy in organ transplantation

**FIGURE 2:**
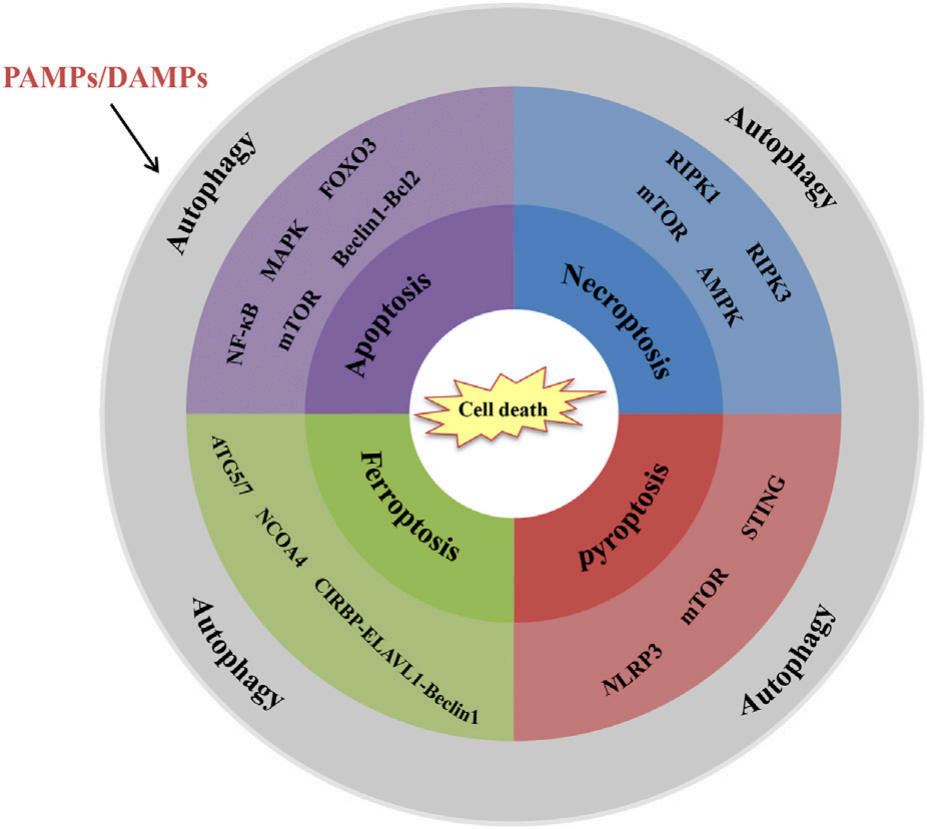
Interrelations between autophagy and cell death in organ transplantation. Schematic shows the key signalling molecules and signalling pathways. (1) NF-κB, MAPK, FOXO3, mTOR and Beclin-1-Bcl2 are involved in autophagy-regulated apoptosis. (2) RIPK1, RIPK3. mTOR and AMPK are involved in autophagy-regulated necroptosis. (3) ATG5/7, NCOA4, and CIRBP-ELAVL1-Beclin-1 axis are involved in autophagy-regulated ferroptosis. (4) NLRP3, mTOR and STING are involved in autophagy-regulated pyroptosis.

The transplant surgery inevitably leads to graft tissue damage and cell death which are closely linked to the activation of innate immune responses. Meanwhile, cell death is also one of the regulatory effects of innate immune responses. Severe cell death of graft tissue will undoubtedly shorten the longevity of the graft and eventually result in failed organ transplants. In the past decades, some studies focused on prolonging graft survival through cell death regulation (Ochando et al., 2019). Under severe stress, autophagy, a highly conserved eukaryotic cellular recycling process, is committed to ensuring cell survival by degrading damaged organelles and toxic metabolites (Qi and Chen, 2019). Growing studies indicate that autophagy is not only a means of keeping cells alive. Also, it is involved in regulating cell death and influences organism immunity (Noguchi et al., 2020). Hence, the potential inter-relational mechanism between autophagy and cell death in organ transplantation is a key component of the innate immune system to the rejection of the transplantation (Figure 2).

### 4.1 The signalling pathway between apoptosis and autophagy during graft transplants

Autophagy and apoptosis both play crucial roles in maintaining homeostasis and are strictly regulated during the cell cycle. Since they share similar activating pathways, they may interact with each other. For example, the kinase inhibitory protein p27 (p27^Kip1^) contributed to decreased pRb expression and increased Bax expression, which are associated with the induction of apoptosis (Fujieda et al., 1999; Naruse et al., 2000). Moreover, p27^Kip1^ can also lead to autophagy during metabolic stress, which seems to protect cells from apoptosis (McKay and White, 2021). There is accumulating evidence indicating that autophagy and apoptosis can interact with each other, either synergistically or antagonistically. Beclin-1 is a key protein that regulates autophagy and apoptosis. VPS34-VPS15-Beclin-1 complex contributes to the extension of autophagic vesicles and the formation of autophagosomes. However, apoptosis-inhibiting molecule Bcl-2 can bind to BH3 of Beclin-1 and form a complex, inhibiting Beclin-1-dependent autophagy (Pattingre et al., 2005). In addition, caspase can mediate the cleavage of Beclin-1, which loses its ability to induce autophagy. And the C-terminal fragment of Beclin-1 will be transferred to mitochondria and amplify mitochondrion-mediated apoptosis (Djavaheri-Mergny et al., 2010). Moreover, mTOR, death-associated protein kinase and Tp53 are all involved in the connection between autophagy and apoptosis (Feng et al., 2018; Yan et al., 2019). During graft transplants, autophagy is rapidly activated after renal ischemia and boosts tubular apoptosis (Jiang et al., 2010). Another study suggested that prolonged autophagy activation might aggravate renal damage by activating the cell death pathway after ischemic kidney injury (Decuypere et al., 2015; Duann et al., 2016). In rat and mouse liver transplant models of hepatic IRI, altered levels of autophagy could regulate hepatocyte apoptosis and protect the liver from warm hepatic IRI under specific circumstances. In addition, Chen et al. found that a strong correlation was observed between the severity of rejection and autophagy levels in CD8^+^ T cells after liver transplantation. The enhanced autophagy aggravates acute graft rejection by suppressing apoptosis of CD8^+^ T cells (Chen et al., 2019). Furthermore, NF-κB signalling, MAPK signalling, FOXO3 signalling and mTOR signalling have been involved in this process, but the specific mechanisms are unclear (Hu et al., 2021). Activation of autophagy protects heart from myocardial ischemia damage and inhibits apoptosis. In contrast, the role of autophagy in myocardial IRI and coronary atherosclerosis remains controversial (Dong et al., 2019). Few studies have discussed the specific regulatory mechanisms involved.

### 4.2 The outcome of the interaction between necrosis and autophagy affects graft survival

Necroptosis is a kind of cell death which is caused by tissue injury or cell inflammation. Although the morphological characteristics of necroptosis and apoptosis are distinct, these two processes are closely related (Park et al., 2021). The interaction between autophagy and necroptosis is more complex. Since autophagy can protect cells by limiting tumour necrosis and inflammatory response, some researchers originally thought activation of autophagy helped inhibit all necroptosis and promote cell survival *via* blocking apoptosis. However, there was evidence that necroptosis could promote autophagy initiation and inhibit the degradation of autophagosomes (Wu W. et al., 2021). In the process of necroptosis, two receptor-interacting protein kinases (RIPK1 (Receptor-interacting protein kinase 1, RIPK1) and RIPK3) are the most important signalling molecules contributing to forming a “necrosome”. Liu et al. reported that the expression of RIPK1 boosted neuron autophagy after traumatic brain injury *via* activation of the NF-κB signalling pathway (Liu J. et al., 2020). Moreover, RIPK1 can promote tuberous sclerosis complex 2 (TSC2) phosphorylation at Ser1387 by AMPK, which will inhibit mTORC1 activity and cause autophagy (Najafov et al., 2021). Recent research confirms that there is an LC3 interacting region domain in the protein sequences of RIPK1 and RIPK3 (Huang et al., 2021). The interaction between LC and RIPK1/3 suggests autophagy-related LC3 accumulation can regulate necroptosis. During human pulmonary IRI, the cold ischemia can increase the Expression of RIPK3 *via* phosphorylation of STAT3 and then cause necroptosis. Treating with a RIPK inhibitor (necrostatin-1) inhibits necrotic cell death (Kim et al., 2018). Similarly, necrostatin-1 can ameliorate primary graft dysfunction by inhibiting RIPK function and necroptosis after rat pulmonary transplantation (Kanou et al., 2018). So we hypothesized that RIPK is a crucial signal molecule that connects autophagy and necroptosis in organ transplants. Further exploring this signalling in the autophagy-necroptosis axis may help prolong graft survival.

### 4.3 Other types of cell death

Some other forms of cell death are still involved in organ transplantation. In a model of coronary artery ligation–induced myocardial IRI, ferroptosis contributes to inflammatory responses and leukocyte trafficking (Li W. et al., 2019). Similar results have also been found in pulmonary IRI (Xu et al., 2020a). The in-depth study shows that autophagy interacts with other cell death forms, such as ferroptosis and pyroptosis. Autophagy boosts ferroptosis *via* the degradation of ferritin. Autophagy-related genes ATG5, ATG7, and nuclear receptor coactivator 4 (NCOA4, which is a selective cargo receptor for ferritinophagy) play key roles in this process (Hou et al., 2016). Mitochondrial ROS is crucial for lipid peroxidation and ferroptosis initiation. Meanwhile, autophagy can regulate ROS in a variety of ways. A recent report indicated that oncogene-induced PI3K-AKT-mTOR signalling activation could inhibit ferroptosis *via* SREBP1/SCD1-mediated lipogenesis (Yi et al., 2020). Since mTOR is one of the most important kinases that regulate autophagy, it may be a key molecule that connects autophagy and ferroptosis. In the process of renal IR, Sui et al. reported that cold-inducible RNA-binding protein (CIRBP) promoted erastin-induced ferroptosis *via* directly interacting with ELAV-like RNA binding protein 1 (ELAVL1), which is considered a critical regulator in the activation of ferroptosis (Sui et al., 2021). Meanwhile, ELAVL1 can also bind to the AU-rich element of Beclin-1, which is one of the most important autophagy regulators (Zhang et al., 2018). Inhibiting autophagy or ELAVL1 can decrease CIRBP-mediated ferroptosis activation (Sui et al., 2021). Therefore, ELAVL1 may be a key molecule that connects ferroptosis and autophagy following organ transplantation. And other studies suggested that pyroptosis might affect primary graft dysfunction. Lin et al. indicated that inhibiting pyroptosis relieves pulmonary IRI (Fei et al., 2020). Recently, some studies have shown the interaction between autophagy and pyroptosis in transplant-associated IRI. As a herbal extract, baicalein is useful in treating IRI. Mechanically, baicalein-mediated autophagy inhibits pyroptosis and endoplasmic reticulum stress, which further alleviates in IRI model (Wu et al., 2020). Another study also reported that autophagy and pyroptosis took part in cerebral IRI together, but the specific mechanism remains unclear (Liu et al., 2021). Since NLRP3, mTOR, and STING concurrently participate in autophagy and pyroptosis, we speculate they may play important roles in regulating graft cell death (Li M. Y. et al., 2019; Liu J. J. et al., 2020; Wu C. et al., 2021; Zhang R. et al., 2021).

## 5 Conclusions and prospects

The success/failure of organ transplantation largely depends on the effective control of graft rejection response. Although many researchers believe that the adaptive immune system is the core factor affecting transplantation rejection, the role of innate immunity in this process is increasingly concerned. The cell receptors can recognize surgical mechanical injury and graft ischemia-reperfusion injury, thus causing inflammation, oxidative stress, cell death and triggering adaptive immunity. Autophagy affects the release of immune mediators by eliminating intracellular toxic substances and then regulates inflammation, oxidative stress, cell death and innate immune signalling pathway. As one of the most commonly used autophagy inducers, rapamycin is widely believed to be effective in alleviating immune rejection after organ transplantation (Tonshoff, 2020). However, since autophagy is a “double-edged sword”, the benefits it brings and its potential threats are two sides of the same coin. A study also shows that chloroquine, as an autophagy inhibitor, prolongs murine skin and heart allograft survival by up-regulating CTLA-4 expression (Cui et al., 2020). Unfortunately, this study did not consider the changes of autophagic flux during the whole process. The dynamics and roles of autophagy in different graft transplants remain controversial and elusive. We hypothesized that different cell types, injury levels, stress intensity and duration might ultimately affect the function of autophagy. It seems that low-level transplant stress will trigger protective autophagy, which contributes to maintaining homeostasis. Under severe transplant stress (such as prolonged ischemia or severe reperfusion injury), however, autophagy always leads to aggravated inflammatory response, oxidative damage, and cell death. In view of the fact that most studies still focus on the observation of autophagy activity during organ transplantation, the specific mechanisms responsible for the interaction between autophagy and innate immunity are still not well understood. In addition, organ transplantation is a complex process that will trigger all kinds of stress, such as mechanical injury, mucosal injury ischemia, hypoxia, hyperoxia and bacterial translocation. However, most studies focus on just one of these factors but ignore the combined effect of these stimuli on autophagy. Therefore, choosing an appropriate experimental model is crucial for us to properly understand the role of autophagy in graft transplants. This review summarizes how autophagy regulates these processes following organ transplantation. Firstly, some important autophagy-related proteins can directly interact with PRRs or the signalling molecule downstream of PRRs, including ATG16L1-NOD1, ATG5/12-RIG-I, TRAF6-Beclin-1, et al. As a result, autophagy is involved in regulating innate immune signal transduction in organ transplantation. Secondly, autophagy regulates inflammation response and oxidative stress by directly degrading pro-inflammatory cytokines and oxidizing intermediates or targeting NLRP3, type II interferon and PI3K signalling during multiple graft transplants. In addition, autophagy contributes to various cell death forms to influence primary graft dysfunction. Therefore, exploring these key programs and signalling molecules will help us reveal how autophagy regulates graft rejection from the perspective of innate immunity.
